# SPP1^+^ Macrophage‐Associated Prognostic Signature in Hepatocellular Carcinoma via Integrated Single‐Cell and Bulk Transcriptomic Analysis

**DOI:** 10.1155/ijog/5555332

**Published:** 2025-09-12

**Authors:** Suyang Yue, Qin Ding, Shanzhong Tan, Pengpeng Zhang

**Affiliations:** ^1^ Department of Integrated TCM and Western Medicine, Nanjing Hospital Affiliated to Nanjing University of Chinese Medicine, Nanjing, China; ^2^ Department of Gastroenterology, Huai’an Second People’s Hospital, The Affiliated Huai’an Hospital of Xuzhou Medical University, Huai’an, China, xzmc.edu.cn; ^3^ Department of Anesthesiology, Huai’an Second People’s Hospital, The Affiliated Huai’an Hospital of Xuzhou Medical University, Huai’an, China, xzmc.edu.cn

**Keywords:** HCC, prognostic signature, single-cell RNA sequencing, SPP1^+^ macrophages, TME

## Abstract

**Background:** Hepatocellular carcinoma (HCC) is a major cause of cancer mortality, with limited treatment options due to its high heterogeneity. SPP1^+^ tumor‐associated macrophages are emerging as key regulators of the tumor immune microenvironment and disease progression.

**Methods:** We integrated scRNA‐seq data from the GEO database with bulk transcriptomic data from TCGA and ICGC. Immune cell subsets were identified through clustering and ligand–receptor interaction analyses. Prognostic genes associated with SPP1^+^ macrophages were screened using univariate Cox and Lasso regression. A risk model was built and validated using survival analysis and ROC curves. A multialgorithm AI framework was applied to enhance model performance.

**Results:** Twelve immune cell types were identified, with SPP1^+^ macrophages showing strong interactions with tumor and immune cells. A seven‐gene signature (SNX5, YBX1, GNPD1, RAB32, TPM3, ATP6V0B, and RAB7A) was constructed, effectively stratifying patients by survival risk in both TCGA and ICGC cohorts. The model showed strong predictive power with high AUC values and a significant correlation between gene expression and risk scores.

**Conclusion:** SPP1^+^ macrophages play a crucial role in HCC immune modulation and progression. The gene signature developed provides a reliable tool for prognosis and may inform personalized treatment. This SPP1^+^ macro‐associated signature offers a novel and robust biomarker for prognosis and may guide precision immunotherapy strategies in HCC.

## 1. Introduction

Hepatocellular carcinoma (HCC) represents a major global health concern and is the third leading cause of cancer‐related mortality worldwide, with its incidence and death rates steadily rising [1, 2]. As the most prevalent form of liver cancer, accounting for approximately 90% of all liver cancer cases, HCC poses a significant threat to public health and imposes a substantial societal burden [3, 4]. Despite notable advances in clinical management over recent decades, surgical resection remains the primary curative option for patients diagnosed at an early stage [5]. However, the exceptionally high rate of postoperative recurrence severely compromises long‐term patient outcomes [6, 7]. Immunotherapy, particularly immune checkpoint inhibitors, has revolutionized systemic treatment across various malignancies [8]. Recent studies have demonstrated that monoclonal antibodies targeting adaptive immune checkpoints such as programmed cell death Protein 1 (PD‐1), programmed death Ligand 1 (PD‐L1), and cytotoxic T‐lymphocyte‐associated Protein 4 (CTLA‐4) can significantly enhance clinical outcomes in HCC [9, 10]. Nonetheless, current immunotherapeutic approaches yield durable responses in only a subset of HCC patients and have yet to substantially improve the prognosis for the majority [11]. Furthermore, HCC is characterized by a high degree of heterogeneity [12]. Therefore, a comprehensive understanding of the molecular mechanisms and immune microenvironment of HCC is urgently needed to develop more precise and effective therapeutic strategies aimed at improving long‐term survival and quality of life for patients.

In HCC, the different subtypes of the tumor microenvironment (TME) have been found to correlate with patient responses to immunotherapy and are essential to tumor progression and treatment effectiveness [13–15]. The TME encompasses various components, including immune cells, stromal cells, and mesenchymal stem cells. Among immune cells, tumor‐associated macrophages (TAMs), dendritic cells, T cells, B cells, and natural killer (NK) cells play critical roles [16]. Previous studies have revealed that TAMs not only interact with HCC tumor cells but also modulate the functions of other immune cells, thereby promoting tumor progression [17].

Secreted Phosphoprotein 1 (SPP1), also known as osteopontin (OPN), is a multifunctional glycoprotein involved in various physiological and pathological processes, including inflammatory responses, immune regulation, and tissue remodeling [18, 19]. Within the oncogenic context, SPP1 is widely recognized for its involvement in tumor progression, invasion, metastasis, and the reprogramming of the TME [20, 21]. Notably, Liu et al. identified that SPP1^+^ macrophages, in concert with cancer‐associated fibroblasts, may constitute a physical immunological barrier within the tumor, restricting immune cell infiltration into the tumor core [22].

Machine learning and deep learning have emerged as powerful tools to address complex medical problems by leveraging large‐scale clinical datasets. These methodologies have consistently demonstrated remarkable performance in predictive modeling and clustering tasks. By employing these advanced analytical techniques, we can explore mechanisms of therapeutic resistance across multiple omic levels—transcriptomics, epigenomics, and translatomics—uncovering insights that may enhance treatment efficacy [23–25]. In this study, we developed a novel artificial intelligence (AI)–based network that integrates conventional regression algorithms, machine learning, and deep learning to construct a gene model associated with SPP1^+^ macrophages. This approach allows for more precise prediction and analysis of clinical outcomes in HCC patients.

Accordingly, the aim of this study is to identify signature genes associated with SPP1^+^ macrophages in HCC and elucidate their prognostic significance. The novel gene model developed in this preliminary investigation demonstrates superior prognostic predictive performance and offers guidance for personalized therapeutic strategies. It also holds promise for advancing SPP1^+^ macrophage‐targeted cancer therapies.

## 2. Methods

### 2.1. Acquisition and Integration of Transcriptomic Data

RNA expression profiles, gene mutation data, and associated clinical information for HCC were retrieved from The Cancer Genome Atlas (TCGA) database (*n* = 1095). The dataset was randomly divided into a training cohort (70%) and a validation cohort (30%). The training set was employed to construct the prognostic model, while the validation set was used to assess its robustness and predictive accuracy. Additionally, transcriptomic data from HCC patients were downloaded from the International Cancer Genome Consortium (ICGC) to serve as an independent external validation cohort. All datasets were formatted as transcripts per million (TPM) and log2‐transformed prior to subsequent analyses. Batch effects between the TCGA and ICGC datasets were corrected using the “SVA” R package.

### 2.2. Acquisition and Analysis of Single‐Cell RNA Sequencing (scRNA‐Seq) Data

A scRNA‐seq dataset of HCC, registered under Accession Number GSE149614 and comprising 10 samples, was obtained from the Gene Expression Omnibus (GEO) database. Quality control was conducted using the “Seurat” and “SingleR” R packages. Cells were retained based on the following criteria: mitochondrial gene content below 10%, total gene count exceeding 200, and gene expression observed in at least three cells with counts between 200 and 7000. To ensure high‐quality data, linear regression modeling and logarithmic normalization were applied for further scaling and standardization. The top 3000 highly variable genes were identified using the FindVariableFeatures function. Canonical correlation analysis (CCA) and the FindIntegrationIntegrators function were employed to mitigate batch effects across samples in downstream analyses. The “IntegrateData” and “ScaleData” functions facilitated comprehensive data integration and normalization. Dimensionality reduction was performed using principal component analysis (PCA), followed by t‐distributed stochastic neighbor embedding (t‐SNE) for visualization. Clustering was executed using FindNeighbors, FindClusters, and RunTSNE with resolutions ranging from 0.1 to 0.5. Based on canonical marker gene expression patterns, cells were annotated into 12 distinct types, including B cells, CD14^+^ monocytes, endothelial cells, hepatocytes, inflammatory macrophages, Kupffer cells, mast cells, myofibroblasts, NK cells, plasma cells, SPP1^+^ macrophages, and T cells. Cell type annotations were primarily based on markers referenced from CellMarker (http://xteam.xbio.top/CellMarker/index.jsp) and CellMarker2.0 (http://bio-bigdata.hrbmu.edu.cn/CellMarker/index.html). The SPP1^+^ macrophage‐associated genes were identified using the “FindMarkers” function from the Seurat R package. The analysis parameters were set as follows: log fold change threshold of 0.25, *p* value threshold of 0.05, and minimum expression percentage (min.pct) of 0.1.

### 2.3. AUCell and Cell–Cell Communication Analysis

Cell–cell interaction networks among various cell types were investigated using the CellChat framework [26]. CellChat enables quantitative inference and analysis of intercellular communication networks from scRNA‐seq data. Ligand–receptor interactions with a *p* value < 0.05 were considered statistically significant and indicative of meaningful communication between cellular subpopulations.

### 2.4. Construction of SPP1^+^ Macrophage‐Associated Risk Signature

Prognostically relevant genes associated with SPP1^+^ macrophage‐associated were initially identified via univariate Cox regression analysis. In line with earlier research [27], developing an optimal SPP1^+^ macrophage‐associated risk signature utilized a comprehensive computational framework that incorporated 10 machine learning techniques: stepwise Cox regression analysis, Lasso regression, Ridge algorithm, plsRcox approach, CoxBoost methodology, random survival forest (RSF) computation, GBM analysis, ENet calculation, SuperPC method, and survival SVM modeling. This integrated analytical pipeline, incorporating 10‐fold cross‐validation protocols, was designed to generate a signature maximizing the concordance index (C‐index) value. This algorithm was used to assign a risk score to each HCC patient. Patients in the TCGA‐HCC cohort were stratified into high‐risk and low‐risk groups based on the median risk score. Survival differences between the two groups were analyzed, and the predictive performance of the model was evaluated.

### 2.5. Survival Analysis and Model Validation

Kaplan–Meier (KM) survival analysis was conducted to compare overall survival (OS) between high‐ and low‐risk groups, with statistical significance determined using the log‐rank test. The predictive accuracy of the prognostic model was assessed using time‐dependent receiver operating characteristic (ROC) curves. The area under the curve (AUC) values for 1‐, 3‐, and 5‐year survival predictions were calculated to evaluate model robustness and predictive capacity.

### 2.6. Data Analysis Software

All statistical analyses and data visualizations were performed using R software (Version 4.1.0) along with relevant bioinformatics packages, including “ggplot2,” “survival,” and “clusterProfiler.” A *p* value less than 0.05 was considered statistically significant.

## 3. Results

### 3.1. Cell Type Characterization and Marker Gene Identification

We obtained a publicly available scRNA‐seq dataset from the GEO database and analyzed cellular populations derived from multiple HCC patients (e.g., HCC01T and HCC02T). The proportions of various cell types within each patient group—including hepatocytes, macrophages, T cells, and endothelial cells—are illustrated in Figure 1a. Using t‐SNE, we visualized the distribution of cellular populations across patient groups to identify cell type–specific marker genes (Figure 1b). These marker genes were presented in a bubble plot format (Figure 1c). Collectively, based on these markers, we performed t‐SNE analysis to display the distribution of 12 distinct cellular subpopulations (Figure 1d). Figure 1e presents a correlation heatmap of different cell types, revealing a strong correlation between SPP1^+^ macrophages and CD14^+^ monocytes.

Figure 1Cell type distribution, clustering, and correlation analysis across different patient groups. (a) A stacked plot illustrates the distribution of various cell types among the patient groups, ranging from HCC01T to HCC10T, providing a clear visualization of the percentage variations in cellular composition across these cohorts. (b) t‐SNE plot showing the clustering of cells from different patients. Each dot represents a single cell, colored by patient group (HCC01T to HCC10T). (c) Bubble plot displaying typical marker genes for each cell cluster. (d) Single‐cell data of liver cancer (different colors represent different cell types). (e) Heatmap depicting the correlation of cell type abundances across patient groups.

**Figure:**
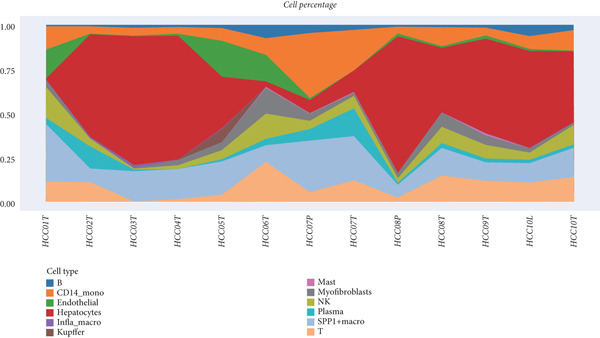
(a)

**Figure:**
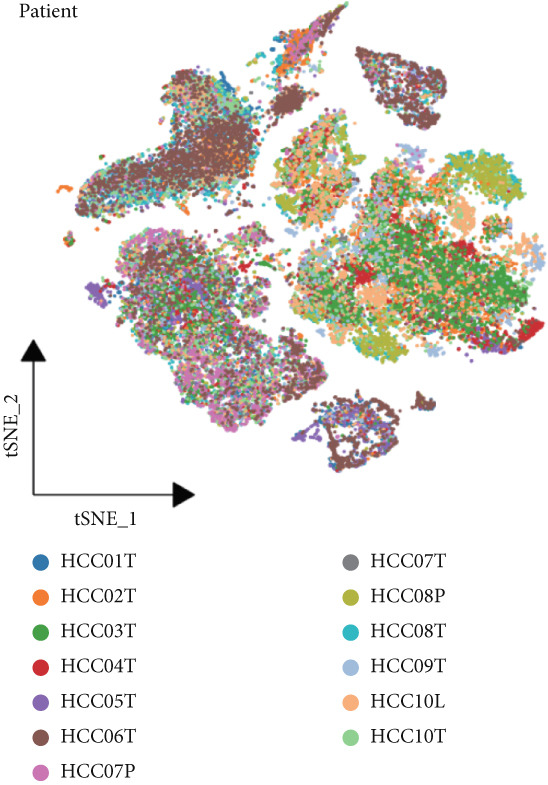
(b)

**Figure:**
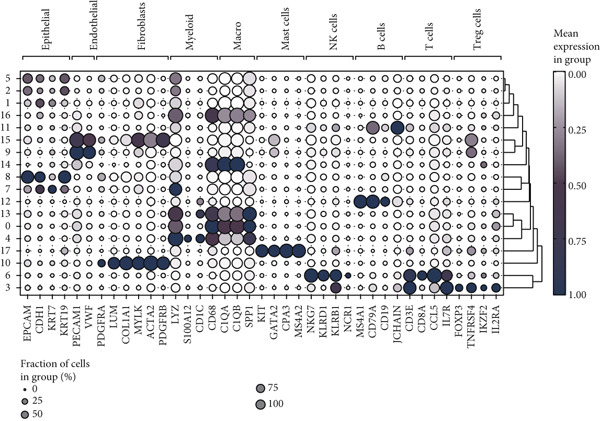
(c)

**Figure:**
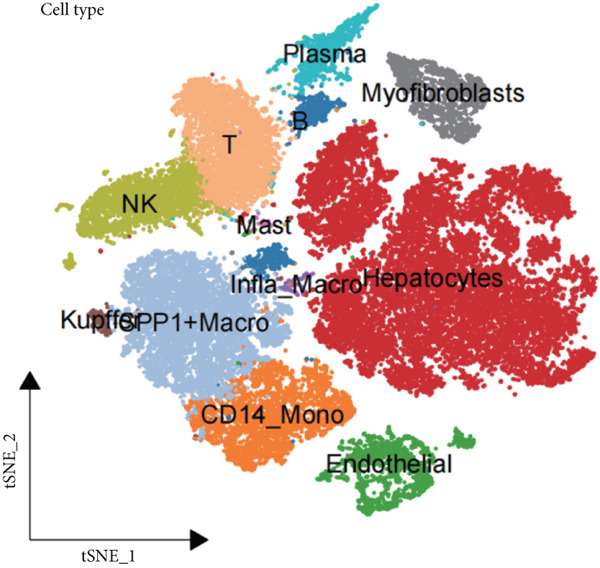
(d)

**Figure:**
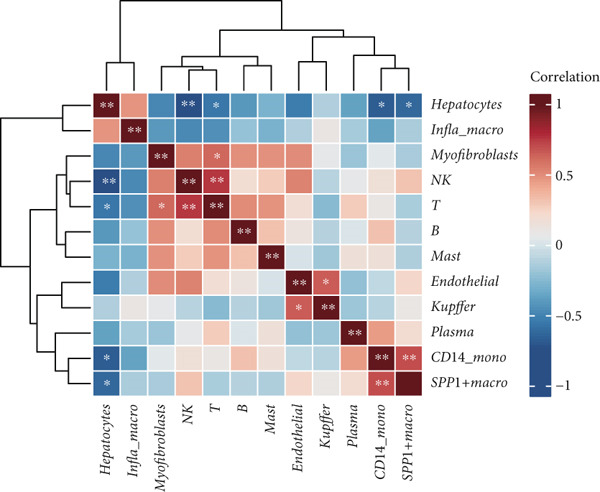
(e)

### 3.2. Single‐Cell Transcriptomic Analysis Reveals Cell–Cell Crosstalk Networks

Figure 2a shows the pathway activity scores across the 12 identified subpopulations. Notably, SPP1^+^ macrophages exhibited pronounced enrichment in pathways associated with immune responses and TME remodeling, suggesting a key immunomodulatory role. To further elucidate intercellular communication and cellular state transitions in HCC, we applied the CellChat package to infer communication networks from scRNA‐seq data (Figure 2b,c). Our results indicated that hepatocytes and immune/stromal cells—such as SPP1^+^ macrophages, T cells, and B cells—exhibited the highest interaction frequencies and interaction strengths (Figure 2d). Additionally, Figure 2e depicts alterations in ligand–receptor interactions between various macrophage subsets and other cell types. SPP1, MIF, and LGALS9 (e.g., LGALS9–CD44 and LGALS9–CD45) signaling pathways were significantly upregulated, indicating that these serve as major output signals from macrophages. These findings underscore the pivotal role of macrophage‐mediated SPP1, MIF, and LGALS9 signaling in HCC pathogenesis.

Figure 2Cell–cell interactions and communication across different cell types. (a) Heatmap showing the signaling pathways and functional correlations between different cell types. (b) Network plot showing the interaction strength (weights) between different cell types. (c) Network plot displaying the number of interactions between different cell types. (d) Pairwise interaction plots between individual cell types. (e) Signaling was identified by comparing the probability of ligand–receptor pair‐mediated communication between different macrophage types and other cell types.

**Figure:**
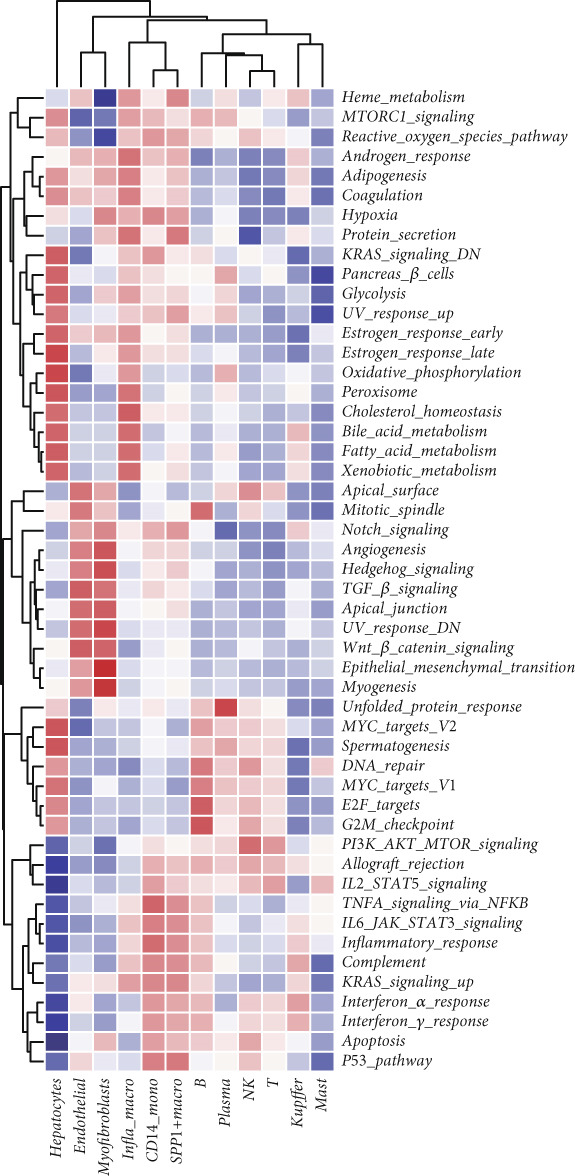
(a)

**Figure:**
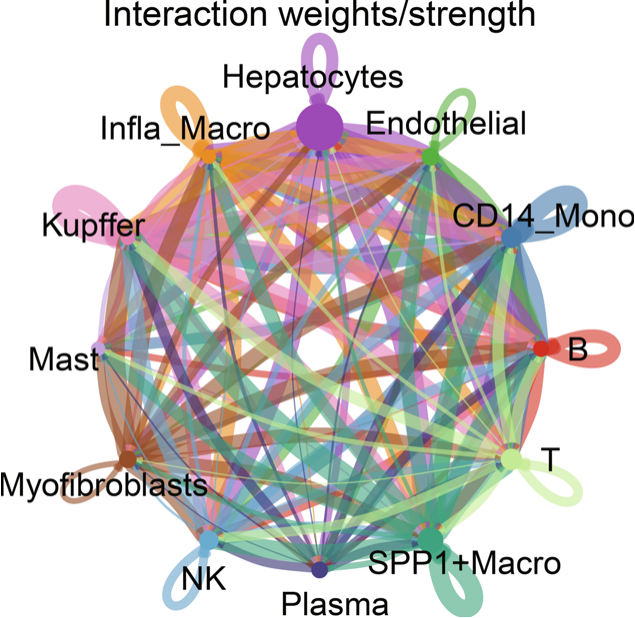
(b)

**Figure:**
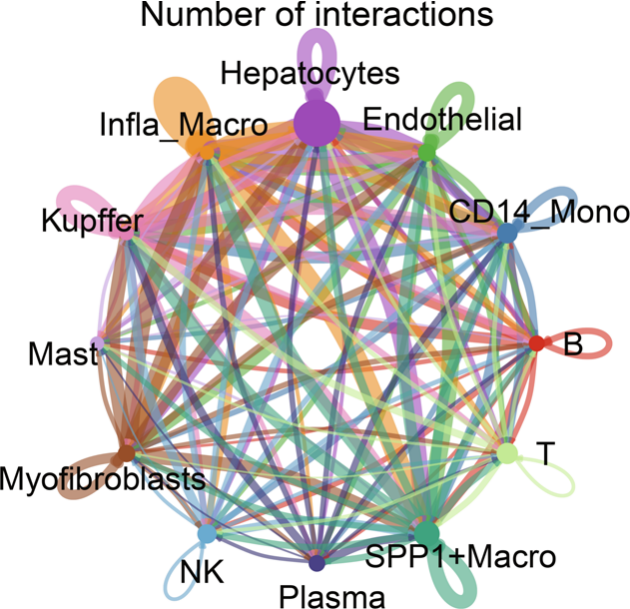
(c)

**Figure:**
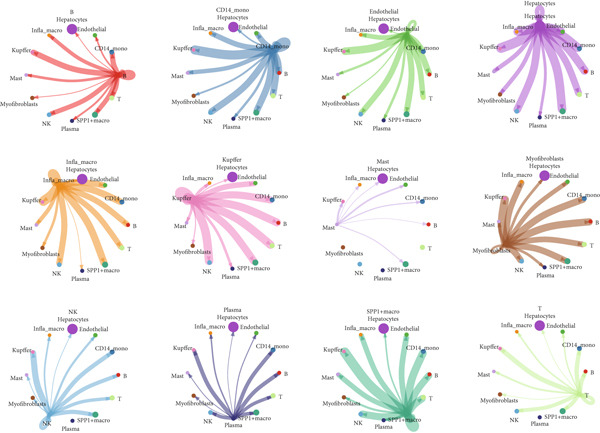
(d)

**Figure:**
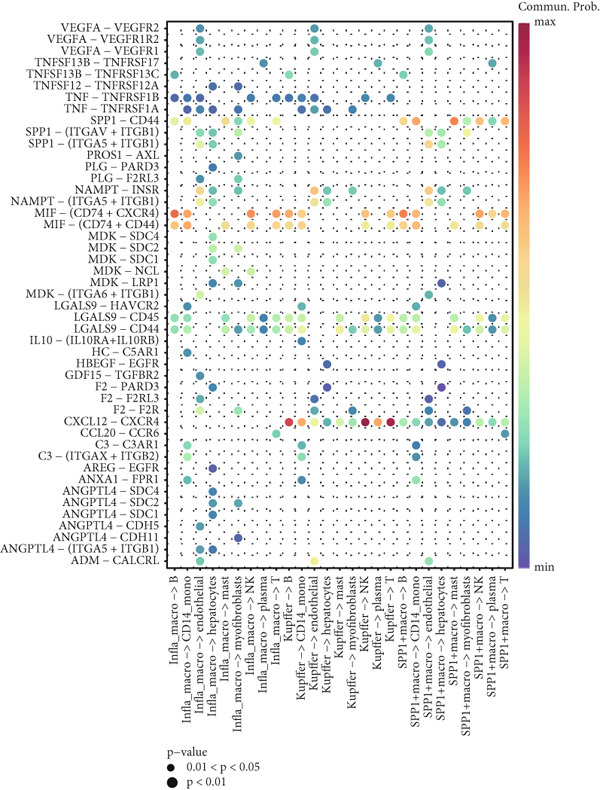
(e)

### 3.3. Identification of SPP1^+^ Macrophage‐Associated Genes

To investigate gene expression profiles and clinical relevance of SPP1^+^ macrophage‐associated genes, we performed a series of bioinformatic analyses. PCA revealed distinct gene expression patterns between samples from the ICGC and TCGA cohorts (Figure 3a,b). Univariate Cox regression identified prognostically significant genes (*p* < 0.01), which also demonstrated differential expression in tumor tissues (|log2FC| > 0.5, adjusted *p* < 0.05). A forest plot (Figure 3c) highlighted key oncogenic factors such as SNX5, YBX1, TPM3, and RAB7A, which were significantly associated with poorer survival and may drive tumor progression.

Figure 3Differential analysis of survival‐related factors and Gene Ontology in TCGA and ICGC datasets. (a, b) PCA plots before and after batch effect removal in the TCGA and ICGC transcriptome dataset. (c) Forest plot showing SPP1^+^ macro‐associated genes. (d) Circos plot illustrating genomic alterations and the distribution of significant genes across chromosomes in the TCGA and ICGC datasets. (e, f) Kyoto Encyclopedia of Genes and Genomes (KEGG) and Gene Ontology (GO) enrichment analyses performed on SPP1^+^ macro‐associated genes.

**Figure:**
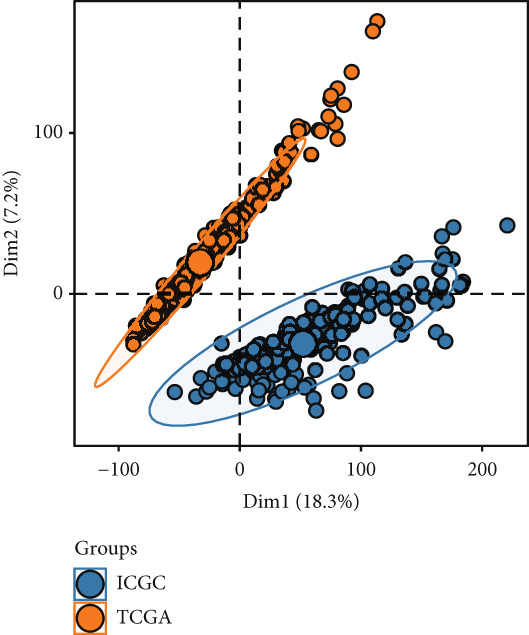
(a)

**Figure:**
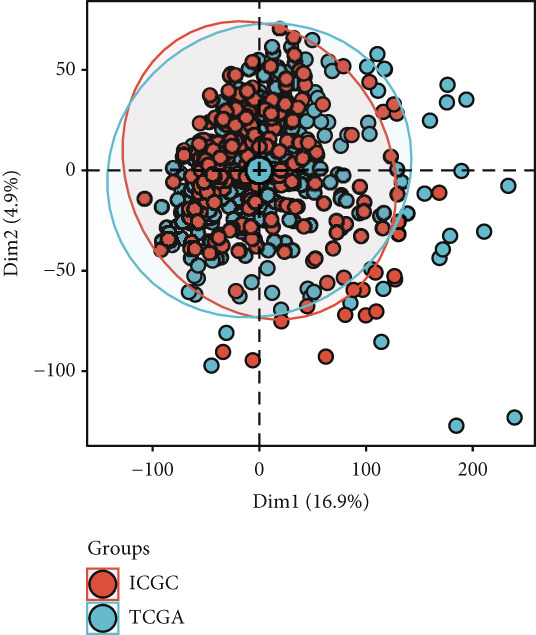
(b)

**Figure:**
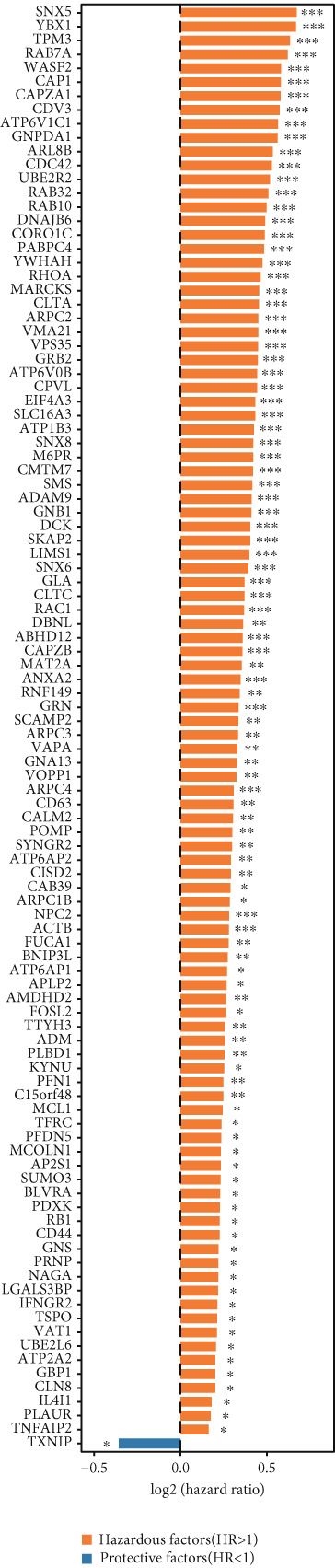
(c)

**Figure:**
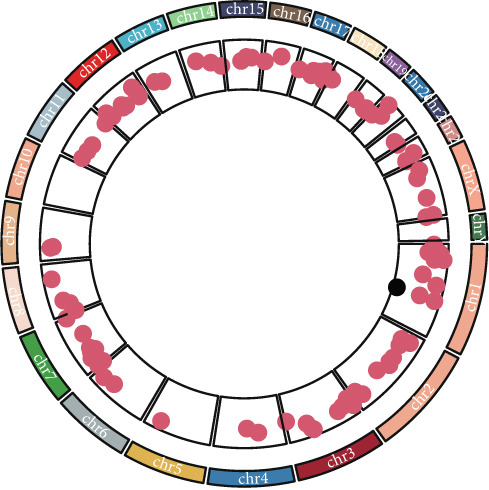
(d)

**Figure:**
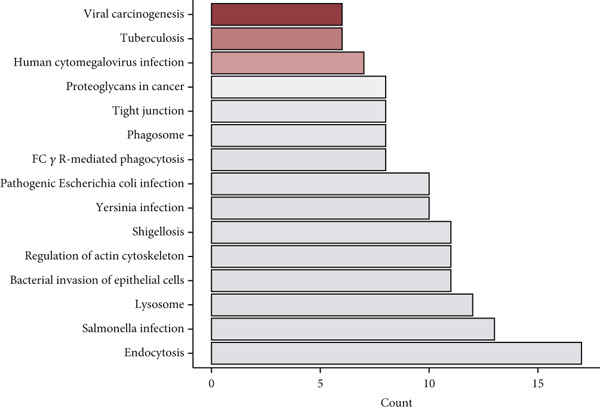
(e)

**Figure:**
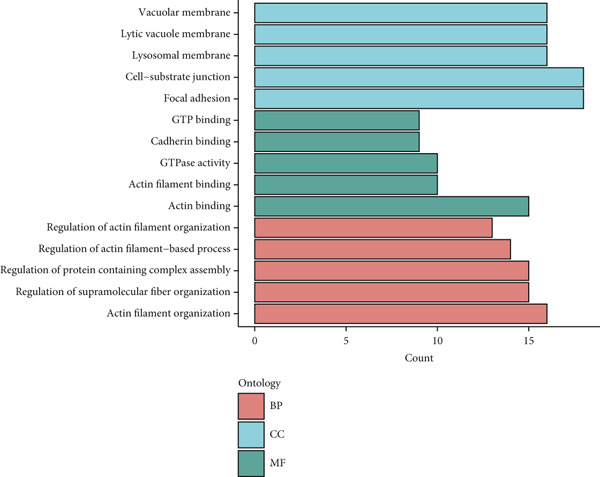
(f)

Pathway enrichment analysis using the Kyoto Encyclopedia of Genes and Genomes (KEGG) revealed these genes were involved in critical biological processes and signaling pathways, including viral carcinogenesis, bacterial invasion of epithelial cells, and cytoskeletal regulation. Gene Ontology (GO) analysis further indicated these genes were enriched in cellular membrane organization, cell–matrix adhesion, and actin cytoskeleton dynamics, suggesting potential therapeutic targets. These results provide biologically relevant insights linking specific genes to HCC prognosis and survival outcomes (Figure 3e,f).

### 3.4. Model Construction and Validation

We evaluated 429 algorithmic combinations across the ICGC and TCGA cohorts and calculated the C‐index for each in the validation dataset. The StepCox (backward) combined with RSF approach achieved the highest average C‐index of 0.828 and was therefore selected as the final SPP1^+^ macrophage‐associated prognostic signature (Figure 4a). Based on the optimal SMRsig score threshold, patients were stratified into high‐ and low‐risk groups. Across all cohorts, patients in the high‐risk group exhibited significantly shorter OS than those in the low‐risk group (*p* < 0.05) (Figure 4c,d).

Figure 4Construction and validation of the signature. (a) Comparison of concordance index (C‐index) values for various algorithm combinations in the two cohorts. (b) Sample size distribution between the two cohorts. (c) Kaplan–Meier survival analysis for SPP1^+^ macro‐associated signature in the TCGA cohort. (d) Kaplan–Meier survival analysis for SPP1^+^ macro‐associated signature in the ICGC cohort.

**Figure:**
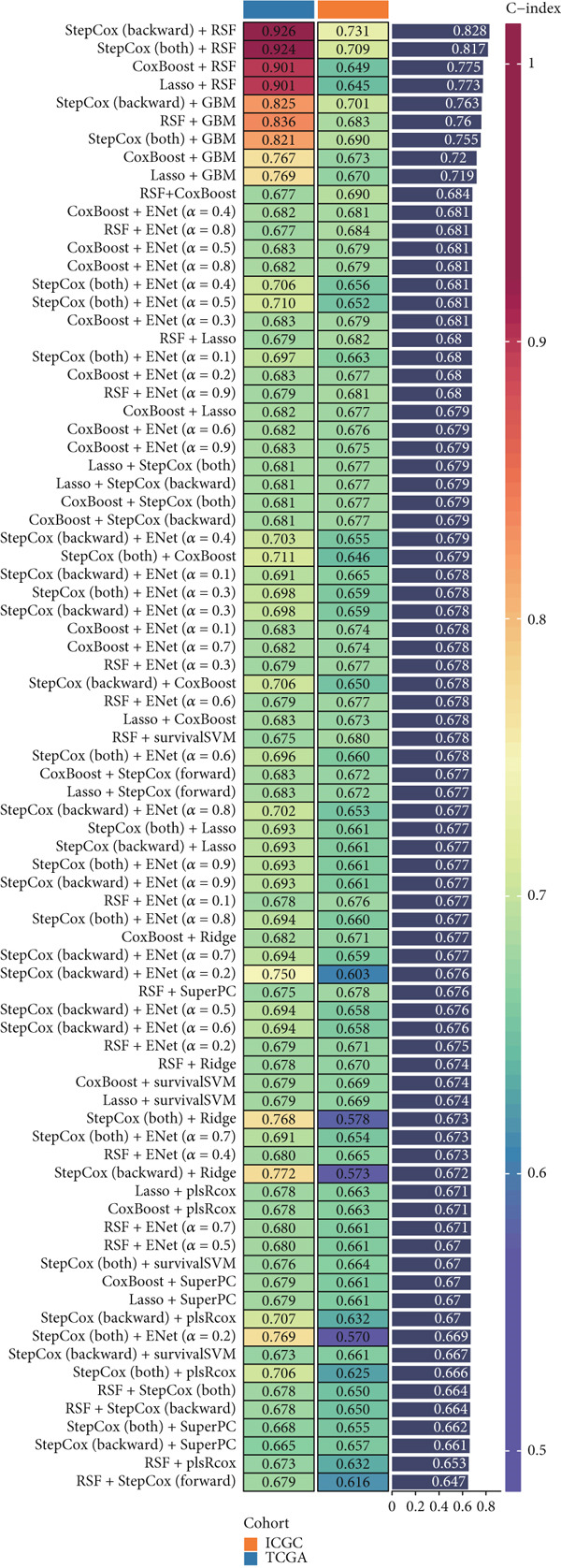
(a)

**Figure:**
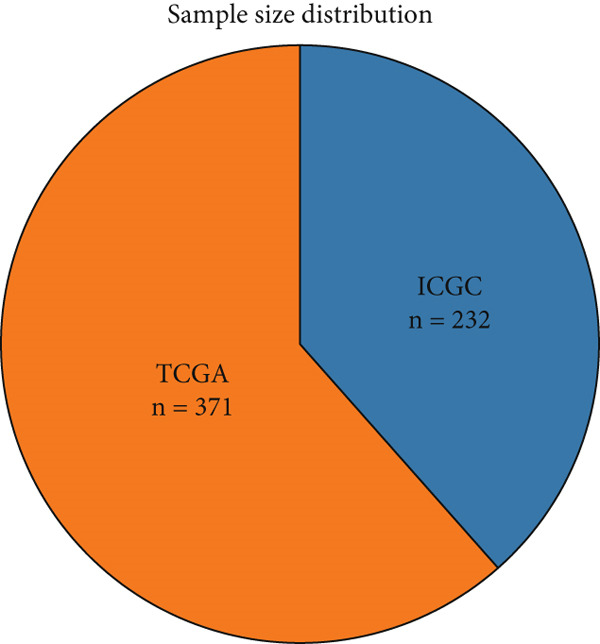
(b)

**Figure:**
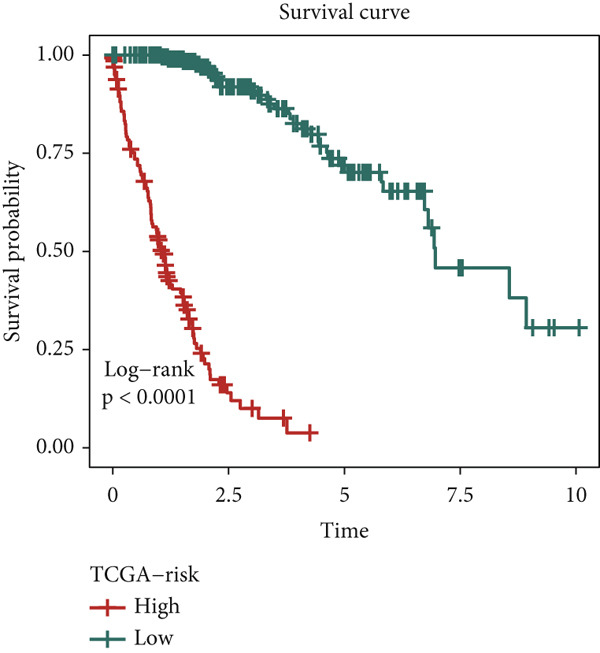
(c)

**Figure:**
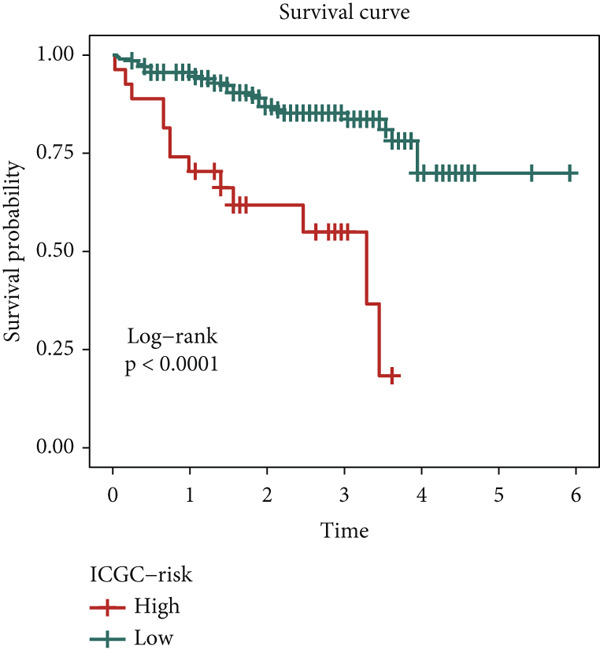
(d)

We next assessed the correlation between the risk score and the expression levels of selected signature genes. Genes such as SNX5, YBX1, GNPD1, RAB32, TPM3, ATP6V0B, and RAB7A showed strong positive correlations with the risk score, with correlation coefficients ranging from 0.49 to 0.72 and *q*‐values of 0 (Figure 5a). Expression profiles effectively distinguished samples across different risk levels, reinforcing the prognostic relevance of these genes (Figure 5b).

Figure 5Correlation analysis and risk score evaluation for survival prediction. (a) Correlation analysis between risk scores and SPP1^+^ macro‐associated genes. (b) PCA plots for the low‐risk (blue) and high‐risk (orange) groups based on the risk score. (c) Area under curve (AUC) values of the SPP1^+^ macro‐associated signature in predicting 1‐, 3‐, and 5‐year survival rates.

**Figure:**
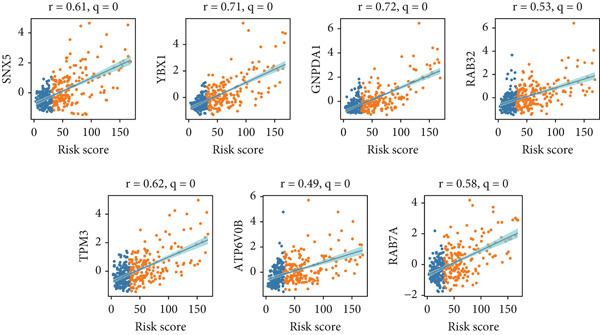
(a)

**Figure:**
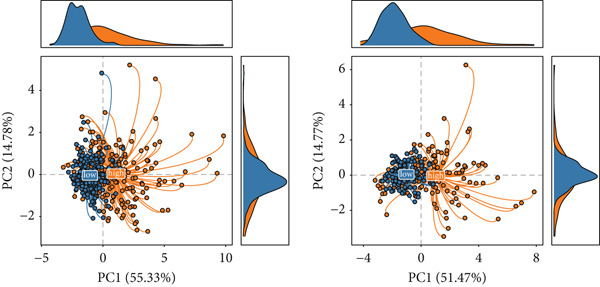
(b)

**Figure:**
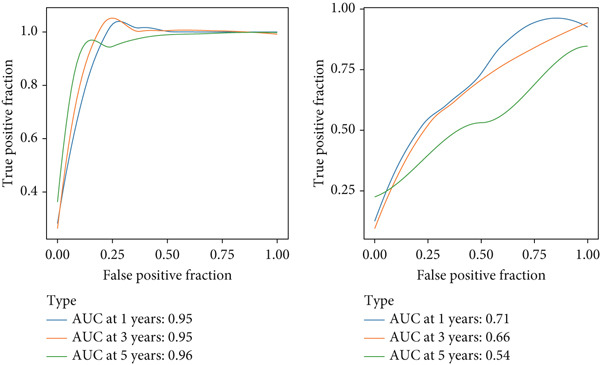
(c)

Finally, the predictive performance of the risk model was validated at 1, 3, and 5 years in both cohorts. The AUC values were 0.95 and 0.64, respectively, indicating high predictive accuracy and robustness (Figure 5c). These results confirm the prognostic utility of the SPP1^+^ macrophage‐associated gene signature and highlight its potential application in clinical risk stratification for HCC.

## 4. Discussion

HCC is characterized by multifactorial etiology and complex molecular mechanisms, with treatment strategies largely dependent on clinical staging [28]. Early diagnosis remains critical for improving therapeutic outcomes [2]. Despite advancements in pharmacological interventions, including targeted therapies and immunotherapies—which have become pivotal in managing advanced HCC—the pronounced tumor heterogeneity limits their efficacy to a subset of patients [29, 30]. This underscores the necessity for a more comprehensive understanding of the tumor immune microenvironment in HCC.

In this study, we leveraged transcriptomic data from scRNA‐seq and conducted in‐depth bioinformatic analyses to elucidate the role of SPP1^+^ macrophage‐associated genes in HCC. We observed that high expression of these marker genes was significantly associated with poor survival outcomes in both TCGA and ICGC cohorts. Utilizing a novel AI‐driven framework, we developed the SPP1^+^ macro‐associated signature to quantify the abundance of SPP1^+^ macrophages and improve prognostic prediction. This model comprises seven genes—SNX5, YBX1, GNPD1, RAB32, TPM3, ATP6V0B, and RAB7A—all of which are implicated in tumor progression, metastasis, and immune evasion [31–36]. Notably, these genes exhibit differential expression in tumor tissues, suggesting their potential as therapeutic targets. Functional enrichment analyses using KEGG and GO revealed that these genes are involved in pathways related to cell membrane dynamics, cell–matrix adhesion, and cytoskeletal organization. These findings provide new insights into mechanisms of HCC progression and offer promising avenues for early diagnosis and targeted therapy.

Given the poor prognosis of advanced HCC, we investigated the determinants of patient survival and identified the prognostic utility of SPP1^+^ macrophages. With the advent of RNA sequencing technologies, clinical laboratories can now detect gene expression signatures predictive of prognosis [37]. Our AI framework, based on the expression profiles of SPP1^+^ macrophage marker genes, integrates 11 algorithms from traditional regression, machine learning, and deep learning approaches. Among these, the combination of StepCox (backward) and RSF yielded the most robust performance. This integrative strategy enabled effective feature selection, model optimization, and generalization, resulting in strong predictive power with high AUC values for 1‐, 3‐, and 5‐year survival. Furthermore, the model demonstrated a significant correlation between risk scores and expression levels of SPP1^+^ macrophage‐associated genes, highlighting its clinical applicability.

Despite the valuable insights gained from scRNA‐seq analysis, the immune microenvironment varies substantially among patients. SPP1^+^ macrophages may exert differential effects depending on the immune subtype, contributing to immune evasion and remodeling of the TME [38]. The research should explore the functional roles of these genes across diverse immune subtypes and further investigate their therapeutic potential in the context of immunotherapy in the future.

In summary, our study reveals the pivotal role of SPP1^+^ macrophages in shaping the immune microenvironment of HCC and introduces a novel prognostic model based on their associated gene signature. This model offers a new perspective for risk stratification and personalized treatment while also identifying promising molecular targets for future therapeutic strategies. Nonetheless, certain limitations remain, including the need for broader external validation and large‐scale clinical trials. Future efforts should aim to refine the model and substantiate its clinical utility through prospective studies. This model may facilitate the development of macrophage‐targeted therapies in HCC and support precision oncology approaches.

## 5. Conclusion

This study reveals the important role of SPP1^+^ macrophages in the immune microenvironment of HCC by integrating scRNA‐seq data with multiple bioinformatic analyses. We developed a gene signature model based on SPP1^+^ macrophages that effectively predicts prognosis in HCC patients and shows strong performance in both the TCGA and ICGC cohorts. The model includes seven key genes—SNX5, YBX1, GNPD1, RAB32, TPM3, ATP6V0B, and RAB7A—which may be involved in tumor growth, immune escape, and microenvironment remodeling and may serve as potential targets for therapy. Our AI‐based model combines several algorithms to improve accuracy and generalizability, providing new tools for risk classification and personalized treatment. However, further validation with larger clinical studies is needed to support its future clinical use.

## Ethics Statement

The authors have nothing to report.

## Consent

The authors have nothing to report.

## Conflicts of Interest

The authors declare no conflicts of interest.

## Author Contributions

Suyang Yue contributed to data analysis, statistical analysis, and manuscript preparation. Qin Ding contributed to data collection and manuscript preparation. Shanzhong Tan conceived the study, supervised the research, and revised the manuscript.

## Funding

No funding was received for this manuscript.

## Data Availability

This study used public data from an online database. The data can be obtained freely from TCGA (https://www.cancer.gov/ccg/research/genome-sequencing/tcga) and ICGC (https://dcc.icgc.org/).
